# Toward a Neurobiology of Unrealistic Optimism

**DOI:** 10.3389/fpsyg.2012.00344

**Published:** 2012-09-12

**Authors:** Punit Shah

**Affiliations:** ^1^Department of Psychology, University of SurreyGuildford, UK

Research spanning three decades has found that human judgment is characterized by unrealistic optimism (or “optimism bias”), the tendency to underestimate the likelihood of negative events and overestimate the likelihood of positive events (Weinstein, 1980). This work has recently garnered much interest, some question its existence (Harris and Hahn, 2011), while others have found support for it by using novel experiments (Massey et al., 2011; Simmons and Massey, 2012). Most recently, attention has turned to investigating the neural underpinnings of this phenomenon (Sharot et al., 2007, 2011). A new study (Sharot et al., 2012) has now shown that optimism bias is increased by up-regulating dopaminergic function via dihydroxy-l-phenylalanine (l-DOPA). Sharot et al. (2012) propose that this process occurs as l-DOPA attenuates belief updating in response to bad news about the future. In light of such evidence, the implications for future research on unrealistic optimism are discussed.

Unrealistic optimism is recognized as one of the major human cognitive biases (Kahneman, 2011). It has been the focus of much research, particularly in social and clinical psychology; here it is argued that optimism bias is not just a pervasive feature of human judgment, but a crucial requirement to guard against depression (Taylor and Brown, 1988). Despite the wealth of research, this work has been scrutinized, as many question (e.g., Moore and Small, 2008) the methods used in the majority of studies where the “comparison approach” is used, i.e., where optimism bias is interpreted by optimistic comparisons of one’s personal risk, relative to the average person. A compelling demonstration of how optimism research may be riddled with statistical artifacts has recently emerged (Harris and Hahn, 2011). In contrast, Sharot et al. (2011) provided a promising, new approach to investigate optimism bias, via the concept of *belief change*. Sharot and colleagues reported that unrealistic optimism persists “in the face of reality” because good (versus bad) news is incorporated significantly more into one’s beliefs of personal risk. They reported that this asymmetric “updating” originates from a prediction error bias which correlates (determined by functional magnetic resonance imaging) with activity in various regions of the frontal cortex. Importantly, on first glance, this method does not appear to suffer from the problems inherent in the comparison approach of optimism research, and their findings have been interpreted as evidence of unrealistic optimism. This has naturally shifted attention towards the neurobiology underlying this phenomenon.

In their new study published in *Current Biology*, Sharot et al. (2012) go further, by demonstrating how the differential updating reported previously (Sharot et al., 2011), is modulated by administering l-DOPA as belief updating for bad news becomes impaired. Sharot et al. used the same belief update task employed in 2011; they asked participants to estimate their likelihood of experiencing negative life events. Subjects were then asked to provide a second estimate for each of the events, but only after being given the actual probability for that event to occur to someone of the same socio-economic background. Optimism bias was measured by the degree to which participants *updated* their personal risk from their first and second estimate; the mean update in response to good news was then compared to the mean update for bad news. The crucial manipulation, however, was administration of l-DOPA during the task. l-DOPA is a pre-cursor for the monoamine neuromodulator dopamine, and when administered orally, it increases dopaminergic activity within the brain. Dopamine was chosen as it is known to modulate reward learning (e.g., Berridge and Robinson, 1998) and therefore, it was expected to either enhance belief updating concerning good news and/or diminish updating in response to the bad news. In order to investigate this, subjects completed the belief update task on two separate days. On each day a different set of 40 negative life events were presented. Employing a double-blind procedure, they were given l-DOPA on one occasion and received a placebo on the second occasion. This order was fully counterbalanced. In addition, a control group of participants were administered Citalopram (a Selective Serotonin Reuptake Inhibitor frequently prescribed for the treatment of depression; Trivedi et al., 2006) instead of l-DOPA. Serotonin was identified as an alternative monoaminergic neuromodulator, given the association between optimism bias and depression (Strunk et al., 2006).

As predicted, up-regulating dopamine function increased optimistic bias; that is, when the degree of belief update was compared across the two conditions (drug versus placebo), it was significantly reduced for bad news (or “undesirable information”) when l-DOPA had been administered. There was no such pattern in the control group who received Citalopram. When update scores were analyzed, a significant Condition (l-DOPA/placebo) × Desirability (desirable/undesirable information) interaction, when coupled with *post hoc* tests, revealed that belief update was not modulated by enhanced learning of desirable information. Rather, when l-DOPA was administered, there was significantly diminished belief updating in response to undesirable information, compared to the placebo condition. Hence, Sharot and colleagues inferred that unrealistic optimism is evident, as negative events are consistently underestimated as a result of selective belief updating.

Sharot and colleagues have furthered our understanding of unrealistic optimism: the shift of optimism research into the realm of cognitive neuroscience is encouraging and the conclusions resulting from this latest study are clear: optimism bias is a robust phenomenon with a neurobiological basis. This supports Simmons and Massey’s (2012) recent suggestion, calling for a shift from “whether optimism is a real phenomenon to when and why it emerges” (p. 5). Indeed, Sharot et al. (2011, 2012) go some way to elucidate the possible mechanisms underlying this phenomenon; however, it seems prudent to examine this new approach of investigating unrealistic optimism with the same rigor as the old comparison approach in future research. Particularly, as the sole use of negative events in optimism studies has been shown to be problematic (see Harris and Hahn, 2011). It is clear, however, the continued use of neuroscientific techniques in this field is an exciting prospect – particularly if we are to better understand optimism bias and its impact on mental health.

## References

[B1] Berridge K. C. Robinson T. E. (1998). What is the role of dopamine in reward: hedonic impact, reward learning, or incentive salience? Brain Res. Brain Res. Rev. 28, 309–369 10.1016/S0165-0173(98)00019-8 9858756

[B2] Harris A. J. L. Hahn U. (2011). Unrealistic optimism about future life events: a cautionary note. Psychol. Rev. 118, 135–154 10.1037/a0020997 21058872

[B3] Kahneman D. (2011). Thinking, Fast and Slow. London: Penguin Books

[B4] Massey C. Simmons J. P. Armor D. A. (2011). Hope over experience: desirability and the persistence of optimism. Psychol. Sci. 22, 274–281 10.1177/0956797610396223 21228135

[B5] Moore D. A. Small D. (2008). “When it is rational for the majority to believe that they are better than average,” in Rationality and social responsibility: Essays in Honor of Robyn Mason Dawes, ed. Krueger J. I. (New York, NY: Psychology Press), 141–174

[B6] Sharot T. Guitart-Masip M. Korn C. W. Chowdhury R. Dolan R. J. (2012). How dopamine enhances an optimism bias in humans. Curr. Biol. 22, 1477–1481 10.1016/j.cub.2012.05.053 22795698 PMC3424419

[B7] Sharot T. Korn C. W. Dolan R. J. (2011). How unrealistic optimism is maintained in the face of reality. Nat. Neurosci. 14, 1475–1479 10.1038/nn.2949 21983684 PMC3204264

[B8] Sharot T. Riccardi A. M. Raio C. M. Phelps E. A. (2007). Neural mechanisms mediating optimism bias. Nature 450, 102–105 10.1038/nature06280 17960136

[B9] Simmons J. P. Massey C. (2012). Is optimism real? J. Exp. Psychol. Gen. 10.1037/a0027405 22329753

[B10] Strunk D. R. Lopez H. DeRubeis R. J. (2006). Depressive symptoms are associated with unrealistic negative predictions of future life events. Behav. Res. Ther. 44, 861–882 10.1016/j.brat.2005.07.001 16126162

[B11] Taylor S. E. Brown J. D. (1988). Illusion and well-being: a social psychological perspective on mental health. Psychol. Bull. 103, 193–210 10.1037/0033-2909.103.2.193 3283814

[B12] Trivedi M. H. Rush A. J. Wisniewski S. R. Nierenberg A. A. Warden D. Ritz L. Norquist G. Howland R. H. Lebowitz B. Mcgrath P. J. Shores-Wilson K. Biggs M. M. Balasubramani G. K. Fava M. (2006). Evaluation of outcomes with citalopram for depression using measurement-based care in STAR*D: implications for clinical practice. Am. J. Psychiatry. 163, 28–40 10.1176/appi.ajp.163.1.28 16390886

[B13] Weinstein N. D. (1980). Unrealistic optimism about future life events. J. Pers. Soc. Psychol. 39, 806–820 10.1037/0022-3514.39.5.806

